# Activation of the Nrf2 Signaling Pathway by Kaempferol Enhances Growth Performance and Antioxidant Capacity in Piglets

**DOI:** 10.1002/fsn3.72439

**Published:** 2026-11-13

**Authors:** Yanhui Liang, Yan Liu, Leqi Tong, Xinyue Fu, Zhijian Du, Jing Zhang, Qinghua Liu

**Affiliations:** ^1^ College of Animal Sciences Fujian Agriculture and Forestry University Fuzhou China

**Keywords:** kaempferol, Nrf2 signaling pathway, oxidative stress, piglets, PK‐15 cells

## Abstract

Kaempferol‐enriched *Sophora Fructus* extract is a promising plant‐derived antioxidant feed additive. However, its effects on growth performance and antioxidant capacity in weaned piglets remain poorly documented. This study aimed to investigate whether dietary supplementation with kaempferol‐enriched *Sophora Fructus* extract improves growth performance and antioxidant status in weaned piglets, and further to explore the underlying mechanism using PK‐15 cell oxidative‐stress model. Twelve weaned piglets (5.14 ± 0.24 kg) were randomly assigned to either a control group (basal diet) or a treatment group (basal diet +400 mg/kg kaempferol‐enriched *Sophora Fructus* extract) for a 35‐day feeding trial. To clarify whether kaempferol exerts antioxidant function via the Nrf2 signaling pathway, an H_2_O_2_‐induced oxidative stress model was established in PK‐15 cells. Adding 400 mg/kg kaempferol‐enriched *Sophora Fructus* extract to the diet significantly improved growth performance by increasing body weight on Day 35 (*p* < 0.05) and reducing feed/gain ratio (F/G) (*p* < 0.05), while significantly boosting systemic antioxidant capacity as evidenced by increased serum T‐AOC, GSH‐Px activity, and Nrf2 and heme oxygenase‐1 (HO‐1) levels (*p* < 0.05) and decreased Reactive Oxygen Species (ROS) levels (*p* < 0.05). In vitro, kaempferol pretreatment effectively alleviated H_2_O_2_‐induced oxidative damage in PK‐15 cells. It activated the Nrf2 signaling pathway by promoting Nrf2 nuclear translocation and upregulating downstream antioxidant genes, including HO‐1 and NAD (PH) quinone dehydrogenase 1 (NQO1). Moreover, such protective effects were abrogated by the Nrf2‐specific inhibitor ML385. Collectively, these results indicate dietary supplementation with 400 mg/kg kaempferol‐enriched *Sophora Fructus* extract has the potential to improve growth performance and antioxidant capacity in weaned piglets. In vitro, kaempferol confers protection against H_2_O_2_‐induced oxidative stress in PK‐15 cells mainly by activating the Nrf2 signaling pathway.

## Graphical abstract

Dietary supplementation with kaempferol effectively improved growth performance and antioxidant capacity in weaned piglets. In vitro, kaempferol promoted Nrf2 nuclear accumulation and upregulated the expression of downstream antioxidant genes, thereby alleviating H_2_O_2_‐induced oxidative stress in PK‐15 cells. These findings indicate that the Nrf2 signaling pathway plays a critical role in mediating the antioxidant effects of kaempferol.

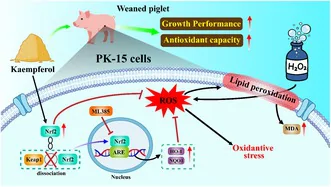

**Abbreviations**
3‐NPA: 3‐nitropropionic acid
ADFI: average daily feed intake
ADG: average daily gain
BW: body weight
CAT: catalase
DMF: dimethyl fumarate
F/G: feed‐to‐gain ratio
FBS: fetal bovine serum
GSH‐Px: glutathione peroxidase
HO‐1: heme oxygenase‐1
iNOS: inducible nitric oxide synthase
Keap1: Kelch‐like ECH‐associated protein 1
MDA: malondialdehyde
ML385: specific small‐molecule Nrf2 inhibitor
NADPH: triphosphopyridine nucleotide
NQO1: NAD (PH) quinone dehydrogenase 1
Nrf2: nuclear factor erythroid 2‐related factor 2
PK‐15 cell: porcine kidney cell
POD: peroxidase
PTE: post‐traumatic epilepsy
ROS: reactive oxygen species
SOD: superoxide dismutase
T‐AOC: total antioxidant capacity

## 1 Introduction

*Styphnolobium japonicum* (L.) Schott (syn. *Sophora japonica* L.) is a medicinal plant widely used in East Asia. Its fruits, commonly referred to as Sophorae Fructus, contain a complex mixture of flavonoids and isoflavonoids, including sophoricoside, rutin, genistein, quercetin‐related compounds, and kaempferol derivatives (Dos Santos et al. 2023; Thabit et al. 2019). Previous studies have demonstrated that fruit‐derived extracts of *S. japonicum* possess free‐radical‐scavenging and antioxidant activities (Elshaarawy et al. 2026; Yang et al. 2025). Kaempferol is an important bioactive flavonol present in this botanical source. The hydroxyl groups at positions 3′, 4′, 5′, and 7′ in the chemical structure of kaempferol can react with free radicals to scavenge them. Therefore, kaempferol modulates cellular physiology through multiple mechanisms, including scavenging reactive oxygen species (ROS), enhancing the antioxidant enzyme activity, and chelating metal ions, which subsequently elevates antioxidant enzyme levels (Alrumaihi et al. 2024; Liao et al. 2016; Yi et al. 2018). Studies have shown that kaempferol inhibits lipid peroxidation induced by NADPH or Fe^2+^ in microsomal systems (Hao et al. 2024). Additionally, it alleviates oxidative damage through its ability to scavenge superoxide anions (Rossi et al. 2025). Consistent with this, one study reported that kaempferol reduces NADPH oxidase activity and F2‐isoprostane levels in rat hippocampal tissues, while elevating the activities of antioxidant enzymes (SOD and CAT), thereby augmenting antioxidant capacity and alleviating Post‐traumatic epilepsy (PTE) in rats (Zhihui et al. 2023). Furthermore, kaempferol attenuates maternal oxidative stress induced by 3‐nitropropionic acid (3‐NPA) via promoting the antioxidant enzyme system, which reduces oxidative damage to fetal offspring and enhances their antioxidant capacity, ultimately exerting organ protective effects (Jian‐hong et al. 2021). Kaempferol also upregulates hemeoxygenase‐1 (HO‐1) expression levels and suppresses the synthesis of nitric oxide (NO) and inducible nitric oxide synthase (iNOS), consequently alleviating LPS‐induced oxidative damage in RAW264.7 cells (Devi et al. 2015; Thi et al. 2023). However, despite extensive evidence supporting the antioxidant properties of kaempferol, its potential as a novel green feed additive for improving antioxidant capacity and growth performance in weaned piglets, along with the underlying mechanisms, remains unclear.

Weaning is a critical period that exerts substantial stress on piglets. Previous studies have shown that weaning stress in piglets readily disrupts the body's oxidative homeostasis, thereby triggering oxidative stress that compromises growth performance and leads to substantial economic losses in swine production (Chen, He, et al. 2025; Chen, Qi, et al. 2025; Liu et al. 2025). This oxidative stress arises from an imbalance between ROS production and the antioxidant defense capacity (Cui et al. 2024). It inflicts oxidative damage on biological macromolecules such as proteins, lipids, and nucleic acids, ultimately leading to cellular dysfunction or cell death (Hui et al. 2022; Xiong et al. 2025). Nrf2, a crucial transcription factor involved in oxidative response regulation, plays a pivotal role in maintaining cellular redox balance and protecting against oxidative injury (Ko et al. 2025). Under normal conditions, Nrf2 is sequestered in the cytoplasm by Kelch‐like ECH‐associated protein 1 (Keap1) (Zhang 2025). Under oxidative challenge, Nrf2 dissociates, translocates to the nucleus, and promotes the transcription of antioxidant genes (e.g., HO‐1, NQO1, SOD) (Bian et al. 2025; Han et al. 2025). Given this pivotal role, exploring the Nrf2 signaling pathway regulators would be a key target to overcome the problem of oxidative stress in weaned piglets.

Therefore, this study aimed to investigate the effects of dietary kaempferol‐enriched *Sophora Fructus* extract supplementation on growth performance and systemic antioxidant capacity in piglets. Furthermore, we utilized PK‐15 cells to establish an in vitro oxidative stress model and to elucidate the role of the Nrf2 pathway in kaempferol's protection. The involvement of this pathway was validated using specific pharmacological activators and inhibitors.

## 2 Materials and Methods

### 2.1 Ethics Consideration

The animal study was reviewed and approved by the Animal Care and Use Committee of Fujian Agriculture and Forestry University (Approval ID: PZCASFAFU24046).

### 2.2 Experimental Materials

A total of 12 healthy crossbred piglets (Duroc × Landrace × Large White) with similar initial body weight (5.14 ± 0.24 kg) were selected from a commercial farm in Wannian County, Jiangxi Province, China. *Sophora Fructus* extract was prepared using an ultrasonic‐assisted extraction method, with its kaempferol content determined by HPLC as 98.2%.

### 2.3 Animals and Housing Conditions

The trial was conducted from April to May at a commercial farm in Wannian County, Jiangxi Province, China. Environmental conditions were maintained at 23°C–26°C and 60%–70% relative humidity throughout the experimental period. All piglets were housed individually in identical pens (1.7 m length × 0.8 m width × 0.8 m height) with the same layout. All piglets were fed 3 times daily at 08:30, 14:30, and 20:30 and had ad libitum access to feed and water throughout the experiment. Prior to the trial, the animal facility was thoroughly cleaned and disinfected by formaldehyde fumigation and iodophor spraying. Routine deworming was also performed. All animals followed the farm's standard vaccination protocol.

### 2.4 Experimental Design

These weaned piglets were randomly divided into 2 treatments with 6 replicates each and 1 piglet per replicate. The control group was fed a basal diet, and the test groups were fed a basal diet supplemented with 400 mg/kg of kaempferol‐enriched *Sophora Fructus* extract daily. The basal diet was formulated to meet the nutrient requirements for weaned piglets as recommended by the NRC (2012). The composition and nutrition level of the experimental diet are presented in Table 1. The experiment commenced at 21 days of age and lasted for 35 days.

**TABLE 1:** Basic diet composition and nutrient levels (%).

Item	21–42 days	43–56 days
Ingredients		
Extruded corn	33.00	10.00
Corn	7.70	39.00
Whey powder	12.50	6.00
Extruded soybean	10.00	8.00
Fermented soybean meal	8.00	8.00
Soybean meal	5.00	11.00
Broken rice	10.00	5.00
Soybean oil	2.50	2.40
Rice bran	2.00	2.50
Coconut oil powder	2.50	1.50
Fish meal	3.00	2.50
Limestone	0.60	0.60
Calcium hydrogen phosphate	1.30	1.20
L‐lysine hydrochloride	0.60	0.60
L‐threonine	0.05	0.20
DL‐methionine	0.10	0.15
Choline chloride	0.10	0.10
Sodium chloride	0.30	0.30
Phytase	0.05	0.05
Acidifier	0.20	0.40
Premix^a^	0.50	0.50
Total	100	100
Analyzed nutrient content^b^		
Digestible energy, MJ/kg	14.59	13.68
Crude protein	17.78	17.51
Crude fat	6.03	4.86
Neutral detergent fiber	6.40	7.29
Acid detergent fiber	2.73	3.09
Ash	4.11	4.34
Total phosphorus	0.54	0.56
Calcium	0.50	0.64
Lysine	1.22	1.33

^a^ The premix supplied the following nutrients per kilogram of the diet: VA 2000 IU, VD_3_ 1000 IU, VE 4 mg, VB_1_ 0.3 mg, VB_2_ 0.8 mg, VB_6_ 0.3 mg, niacin 8 mg, D‐pantothenic acid 4 mg, folic acid0.4 mg, biotin 0.01 mg, Fe 80 mg, Cu 30 mg, Zn 22 mg, Mn 40 mg.

^b^ The digestible energy (DE) was a calculated value, whereas the remaining components were determined through direct measurement.

### 2.5 Growth Performance Measurement

Piglets were individually weighed on Day 0, 21, and 35 of the trial. The daily feed intake was recorded in replicates, and the total feed consumption was summarized at the trial conclusion. Subsequently, average daily gain (ADG), average daily feed intake (ADFI), and feed/gain ratio (F/G) of piglets in each group were calculated.

### 2.6 Sample Collection

Blood samples were collected from all piglets via jugular vein on the final day of the trial. Serum samples were centrifuged at 3000 × *g* for 10 min at 4°C. Then, the supernatant was aliquoted into 2–3 pre‐labeled 1.5 mL microcentrifuge tubes and stored at −80°C for subsequent analysis.

### 2.7 Assay of Serum Antioxidant Indices

Serum levels of total antioxidant capacity (T‐AOC), catalase (CAT), malondialdehyde (MDA), superoxide dismutase (SOD), glutathione peroxidase (GSH‐Px), and peroxidase (POD) were measured using a multifunctional microplate reader. Reactive oxygen species (ROS) were detected by the dihydroethidium (DHE) fluorescent probe assay. Nrf2, HO‐1, and Keap1 levels were quantified via enzyme‐linked immunosorbent assay (ELISA). All experimental procedures strictly followed the manufacturers' protocols of the respective kits and were performed on a microplate analyzer (Rayto RT‐6100) to ensure data accuracy.

### 2.8 Cell Culture

The PK‐15 cell line (porcine kidney epithelial cells) was procured from Wuhan Pricella Biotechnology Co. Ltd. (China). Cells were cultured according to the manufacturer's instructions. The culture medium consisted of 90% DMEM (Dulbecco's Modified Eagle Medium), 10% FBS (Fetal Bovine Serum), and 1% penicillin–streptomycin. Cells were maintained at 37°C in a humidified atmosphere containing 5% CO_2_, and the medium was replaced every 24 h.

### 2.9 Cell Treatments

To investigate whether kaempferol exerts its antioxidant effects through the Nrf2 pathway in vitro, a dual‐intervention experimental design was implemented using an Nrf2 inhibitor ML385 and the Nrf2 activator dimethyl fumarate (DMF). ML385 is a specific small‐molecule Nrf2 inhibitor that binds to the Neh1 DNA‐binding domain of Nrf2 and interferes with its binding to antioxidant response element sequences, thereby suppressing Nrf2‐dependent transcription. In contrast, DMF is an electrophilic Nrf2 activator that modifies cysteine residues of Keap1, thereby reducing Keap1‐mediated Nrf2 degradation and promoting Nrf2 stabilization, nuclear translocation, and downstream antioxidant gene expression. For the Nrf2 inhibition experiment, PK‐15 cells were divided into the following groups: (C) control group without intervention; (M) ML385 group, treated with 10 μM ML385; (OS) oxidative stress group, treated with 1 mM H_2_O_2_; (K + OS) kaempferol protection group, co‐treated with 50 μM kaempferol and 1 mM H_2_O_2_; and (K + OS+M) combination inhibition group, co‐treated with 50 μM kaempferol, 1 mM H_2_O_2_, and 10 μM ML385. For the Nrf2 activation experiment, cells were assigned to the following groups: (C) control group without intervention; (D) DMF group, treated with 50 μM DMF; (OS) oxidative stress group, treated with 1 mM H_2_O_2_; (K + OS) kaempferol protection group, co‐treated with 50 μM kaempferol and 1 mM H_2_O_2_; and (K + OS+D) combination activation group, co‐treated with 50 μM kaempferol, 1 mM H_2_O_2_, and 50 μM DMF.

### 2.10 Quantitative Real‐Time PCR

PK‐15 cells in the logarithmic growth phase were seeded in a 6‐well plate. When the cell confluence reached 60%–70%, the cells were collected after group intervention. RNA was extracted using the Golden Trizol UP reagent kit. Reverse transcription was performed according to the instructions of the One‐Step gDNA Removal and cDNA Synthesis SuperMix kit. The mRNA expression levels of *Nrf2*, *HO‐1*, *NQO1*, and *Keap1* were detected using the SYBR Premix Ex TaqTM real‐time fluorescent quantitative PCR (qRT‐PCR) kit. The primer sequences used in this study are listed in Table 2. β‐actin was used as the internal reference gene. The relative mRNA expression levels of each target gene were calculated using the $2^{-\Delta \Delta C_{t}}$ method in qRT‐PCR results.

**TABLE 2:** Primer sequences for qRT‐PCR.

Gene	Primer sequences (5′ → 3′)	GenBank ID
*β‐Actin*	F: AGAGCAAGGAGGCATCCTG	ON164673
	R:CACGCAGCTCGTTGTAGAAG	
*Nrf2*	F: CAAGTCCAGAAACCAAACCG	XM_021075133
	R:GTGTTTCCTGTTGCGTATG	
*Keap1*	F:ACCCAATTTCTGCCCCTGAG	XM_021076667
	R:ACTTGACCTGCAGCGTAACA	
*HO‐1*	F:CACTCACAGCCCAACAGCAT	NM_001004027
	R:TACAAGGACGCCATCACCAG	
*NQO1*	F:TTTCTGCCTTCCTTGACTTG	NM_001159613
	R:CTTTCCCGGCTTTACATCCT	

*Note:* Primer sequences are shown in the 5′–3′ direction.

Abbreviations: *β‐actin* was used as the reference gene; F, forward primer; *HO‐1*, heme oxygenase‐1; *Keap1*, Kelch‐like ECH‐associated protein 1; *NQO1*, NAD(P)H quinone dehydrogenase 1; *Nrf2*, nuclear factor erythroid 2‐related factor 2; R, reverse primer.

### 2.11 Western Blot

PK‐15 cells in the logarithmic growth phase were seeded in a 6‐well plate. When the cell confluence reached 60%–70%, the cells were collected after group intervention. Total protein was extracted using RIPA lysis buffer, and protein quantification was performed using the BCA method. The proteins were separated by 10% SDS‐PAGE and transferred to a PVDF membrane. The membrane was blocked with 5% skim milk at room temperature for 1 h, followed by incubation with Nrf2 antibody (1:1000) and β‐actin antibody (1:2000) at 4°C for 12 h. After washing with TBST, the membrane was incubated with HRP‐conjugated goat anti‐rabbit IgG secondary antibody (1:8000) at room temperature for 45 min. After washing with TBST, the ECL solution was added for chemiluminescence, and the image was captured using an imaging system. The relative expression levels of the target proteins were calculated by normalizing their band gray values to those of β‐actin, which served as the internal control. The gray values were quantified using ImageJ software.

### 2.12 ELISA

PK‐15 cells in the logarithmic growth phase were seeded into 6‐well plates and collected after intervention according to the grouping when the cell fusion rate reached 60%–70%. The cell pellets were collected and lysed using a cell lysis buffer to extract cytoplasmic proteins. After centrifugation at 4°C, the supernatant containing the cytoplasmic proteins was collected. The pellet was resuspended in the nuclear protein extraction reagent. After incubation on ice, the sample was centrifuged at 4°C, and the resulting supernatant was collected as the nuclear protein fraction. Nuclear Nrf2 content was measured using an ELISA kit according to the manufacturer's instructions.

### 2.13 Statistical Analysis

The data were collated using WPS Office statistical software. Data were analyzed using SPSS version 27.0. For the in vivo experiment, the individual piglet was considered the experimental unit, with 6 experimental units per treatment. Differences between the 2 dietary treatment groups were analyzed using a 2‐tailed independent‐samples *t*‐test. For the in vitro cell experiments involving multiple treatment groups, data were analyzed using one‐way analysis of variance (ANOVA), followed by Duncan's multiple‐range test. Lowercase letters should be used as superscripts, with the same letter indicating no significant difference between groups (*p* > 0.05), and different letters indicating a significant difference (*p* < 0.05).

## 3 Results

### 3.1 Growth Performance

The effects of kaempferol‐enriched *Sophora Fructus* extract on the growth performance of weaned piglets are presented in Table 3. At the beginning of the trial, there was no significant difference in the initial BW among piglet groups (*p* > 0.05). Compared with the control group, dietary supplementation with 400 mg/kg of kaempferol‐enriched *Sophora Fructus* extract exhibited significantly higher BW on Day 35 (*p* < 0.05), but there was no significant change in ADFI and ADG (*p* > 0.05). Moreover, F/G of test groups remained significantly lower than that of controls during the entire experimental period (*p* < 0.05).

**TABLE 3:** Effect of kaempferol‐enriched *Sophora Fructus* extract on the growth performance of weaned piglets.

Item	Times	Control	Treatment	*p*
BW, kg	0 day	5.14 ± 0.27	5.14 ± 0.23	0.98
	21 days	8.70 ± 0.35	9.07 ± 0.57	0.20
	35 days	14.09 ± 0.55^b^	14.84 ± 0.58^a^	0.04
ADG, g	1–21 days	169.76 ± 13.44	187.54 ± 20.42	0.11
	22–35 days	384.64 ± 54.18	412.14 ± 50.10	0.38
	1–35 days	255.71 ± 17.44	277.38 ± 17.24	0.06
ADFI, g	1–21 days	270.56 ± 7.25	268.49 ± 6.62	0.62
	22–35 days	544.52 ± 28.06	532.26 ± 28.01	0.47
	1–35 days	380.14 ± 13.45	374 ± 14.16	0.46
F/G	1–21 days	1.60 ± 0.14	1.45 ± 0.16	0.10
	22–35 days	1.45 ± 0.29	1.35 ± 0.17	0.28
	1–35 days	1.49 ± 0.14^a^	1.36 ± 0.09^b^	0.04

*Note:* Piglets were assigned to a control group receiving the basal diet or a treatment group receiving the basal diet supplemented with 400 mg/kg kaempferol‐enriched *Sophora Fructus* extract (*n* = 6 per group). Data are presented as mean ± SD. Within the same row, values with different lowercase superscript letters differ significantly (*p* < 0.05).

Abbreviations: ADFI, average daily feed intake; ADG, average daily gain; BW, body weight; F/G, feed‐to‐gain ratio.

### 3.2 Antioxidant Capacity

According to Table 4, dietary supplementation with 400 mg/kg kaempferol‐enriched *Sophora Fructus* extract significantly increased activity of T‐AOC and GSH‐Px in serum and levels of HO‐1 and Nrf2 in serum (*p* < 0.05), while it significantly decreased serum ROS (*p* < 0.05). However, there was no significant difference in SOD, MDA, POD, CAT, and Keap1 in serum (*p* > 0.05).

**TABLE 4:** Effects of kaempferol‐enriched *Sophora Fructus* extract on serum antioxidant indices in weaned piglets.

Item	Control	Treatment	*p*
ROS (RFU/mg)	694.16 ± 21.31^a^	659.46 ± 23.94^b^	0.02
SOD (U/mL)	49.10 ± 1.41	50.51 ± 2.74	0.29
CAT (U/mL)	68.81 ± 11.84	66.26 ± 10.45	0.70
T‐AOC (U/mL)	5.81 ± 0.17^b^	6.02 ± 0.08^a^	0.02
MDA (nmol/mL)	1.69 ± 0.12	1.65 ± 0.22	0.70
GSH‐Px (nmol/min/mg)	57.55 ± 5.86^b^	65.64 ± 5.70^a^	0.04
POD (U/mL)	276.85 ± 22.93	303.15 ± 59.24	0.33
HO‐1 (U/mL)	40.08 ± 3.69^b^	44.29 ± 1.89^a^	0.03
Nrf2 (pg/mL)	624.95 ± 33.72^b^	683.62 ± 41.86^a^	0.02
Keap1 (pg/mL)	869.37 ± 55.29	810.44 ± 33.20	0.05

*Note:* Piglets were assigned to a control group receiving the basal diet or a treatment group receiving the basal diet supplemented with 400 mg/kg kaempferol‐enriched *Sophora Fructus* extract (*n* = 6 per group). Data are presented as mean ± SD. Within the same row, values with different lowercase superscript letters differ significantly (*p* < 0.05).

Abbreviations: CAT, catalase; GSH‐Px, glutathione peroxidase; HO‐1, heme oxygenase‐1; Keap1, Kelch‐like ECH‐associated protein 1; MDA, malondialdehyde; Nrf2, nuclear factor erythroid 2‐related factor 2; POD, peroxidase; ROS, reactive oxygen species; SOD, superoxide dismutase; T‐AOC, total antioxidant capacity.

### 3.3 Expression of Genes Associated With the Nrf2 Signaling Pathway

When Nrf2 activity was inhibited by ML385, the protective effects of kaempferol were attenuated, as shown in Figure 1. Compared to the control, the ML385‐treated group showed significantly decreased expression of *Keap1* and *NQO1*, while the H_2_O_2_‐treated group showed significantly decreased *NQO1* expression (*p* < 0.05). Compared with the H_2_O_2_‐treated group, the K + OS group showed a significant increase in the expression of *Nrf2* and *NQO1* and a significant decrease in *Keap1* expression (*p* < 0.05). Compared with the K + OS group, the K + OS+M group showed significantly decreased expression of *HO‐1* and *NQO1* (*p* < 0.05).

**FIGURE 1:**
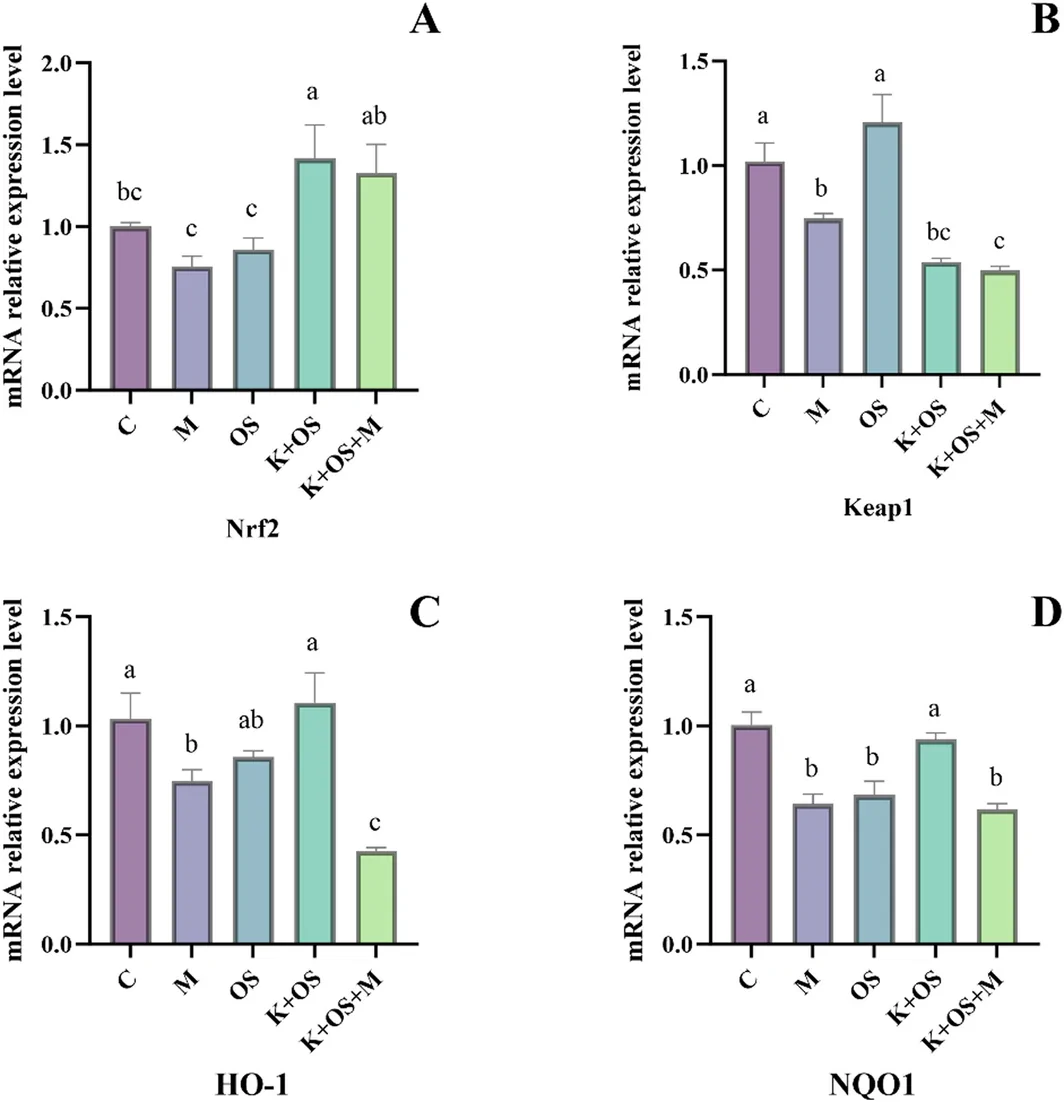
The effect of kaempferol on Nrf2 pathway related genes after Nrf2 inhibitor treatment. (A) Gene expression of *Nrf2*; (B) Gene expression of *Keap1*; (C) Gene expression of *HO‐1*; (D) Gene expression of *NQO1*. PK‐15 cells were seeded in 6‐well plates. The cells were then pretreated with 10 μM ML385 or its vehicle for 24 h, followed by a 24 h incubation with 50 μM kaempferol or its vehicle. Finally, the cells were treated with 1 mM H_2_O_2_ for 3 h. The results are presented as the mean ± SE, *n* = 6. Values without common letters (a–c) differ significantly (*p* < 0.05). *HO‐1*, heme oxygenase‐1; K, kaempferol; *Keap1*, Kelch‐like ECH‐associated protein 1; M, ML385; *NQO1*, NAD (PH) quinone dehydrogenase 1; *Nrf2*, nuclear factor erythroid 2‐related factor 2; OS, hydrogen peroxide.

Inversely, when DMF was used to stimulate Nrf2 activities, the results are shown in the Figure 2. DMF treatment resulted in a significant upregulation of *Nrf2* and *HO‐1* expression and a significant downregulation of *Keap1* expression (*p* < 0.05). H_2_O_2_‐treatment resulted in a significant downregulation of *Nrf2*, *HO‐1*, and *NQO1* and a significant upregulation of *Keap1* (*p* < 0.05). *Keap1* expression was significantly lower in the K + OS group than in the H_2_O_2_‐treated group (*p* < 0.05). Compared with the K + OS group, the K + OS+D group showed significantly increased expression of *Nrf2*, *HO‐1*, and *NQO1* and significantly decreased expression of *Keap1* (*p* < 0.05).

**FIGURE 2:**
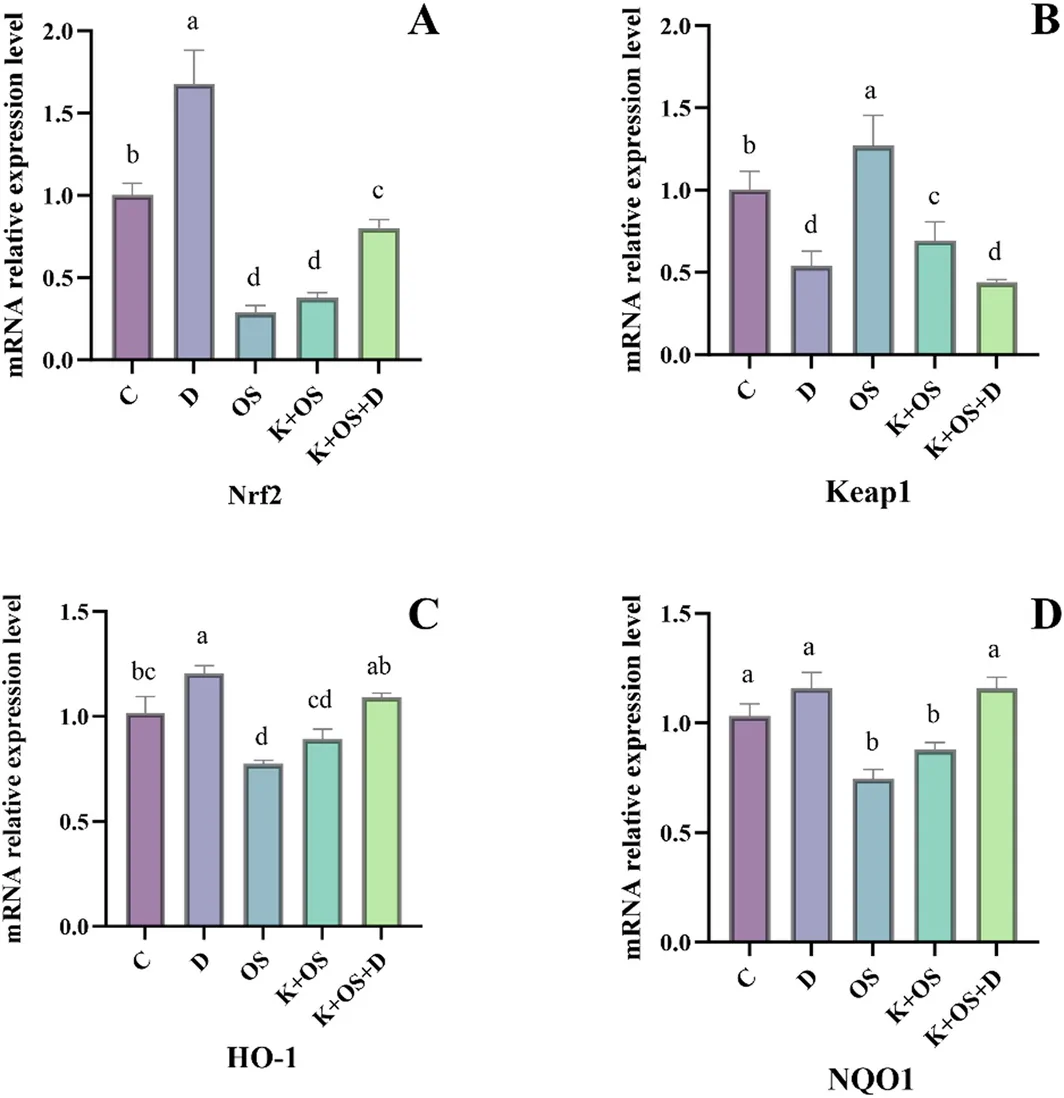
The effect of kaempferol on Nrf2 pathway related genes after Nrf2 activator treatment. (A) Gene expression of *Nrf2*; (B) Gene expression of *Keap1*; (C) Gene expression of *HO‐1*; (D) Gene expression of *NQO1*. PK‐15 cells were seeded in 6‐well plates and subjected to the following sequential treatments: Pretreatment with 50 μM DMF (or vehicle) for 24 h, incubation with 50 μM kaempferol (or vehicle) for a further 24 h, and finally exposure to 1 mM H_2_O_2_ for 3 h. The results are presented as the mean ± SE, *n* = 6. Values without common letters (a–c) differ significantly (*p* < 0.05). D, DMF; *HO‐1*, heme oxygenase‐1; K, kaempferol; *Keap1*, Kelch‐like ECH‐associated protein 1; *NQO1*, NAD (PH) quinone dehydrogenase 1; *Nrf2*, nuclear factor erythroid 2‐related factor 2; OS, hydrogen peroxide.

### 3.4 Expression of Proteins Associated With the Nrf2 Signaling Pathway

When ML385 was used to inhibit Nrf2 activities, the results are shown in Figure 3. Compared with the control group, the Nrf2 protein expression levels in both cytoplasm and nucleus were significantly decreased in the ML385‐treated group and the H_2_O_2_‐treated group (*p* < 0.05). Compared with the H_2_O_2_‐treated group, the K + OS group showed significantly increased Nrf2 protein expression in both cytoplasm and nucleus (*p* < 0.05). In contrast, the K + OS+M group exhibited significantly reduced Nrf2 protein expression in both cytoplasm and nucleus compared to the K + OS group (*p* < 0.05).

**FIGURE 3:**
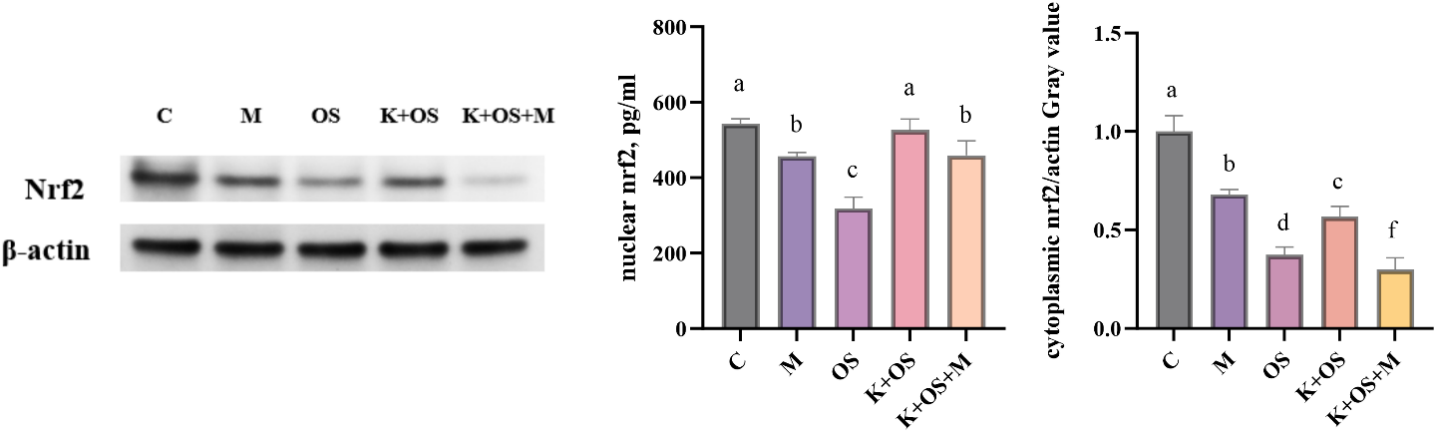
The effect of kaempferol on Nrf2 protein in the nucleus and cytoplasm of PK‐15 cells after Nrf2 inhibitor treatment. After being seeded in 6‐well plates, PK‐15 cells were pretreated with 10 μM ML385 (or vehicle) for 24 h, incubated with 50 μM kaempferol (or vehicle) for a further 24 h, and finally exposed to 1 mM H_2_O_2_ for 3 h. The results are presented as the mean ± SE, *n* = 6. Values without common letters (a–c) differ significantly (*p* < 0.05). K, kaempferol; M, ML385; OS, hydrogen peroxide.

Inversely, when DMF was used to stimulate Nrf2 activities, the results are shown in Figure 4. Compared with the control group, the Nrf2 protein expression levels in both cytoplasm and nucleus were significantly increased in the DMF‐treated group and decreased in the H_2_O_2_‐treated group (*p* < 0.05). Compared with the H_2_O_2_‐treated group, the K + OS group showed significantly increased Nrf2 protein expression in both cytoplasm and nucleus (*p* < 0.05). In contrast to the K + OS group, the K + OS+D group showed significantly reduced cytoplasmic Nrf2 protein levels but increased nuclear Nrf2 levels (*p* < 0.05).

**FIGURE 4:**
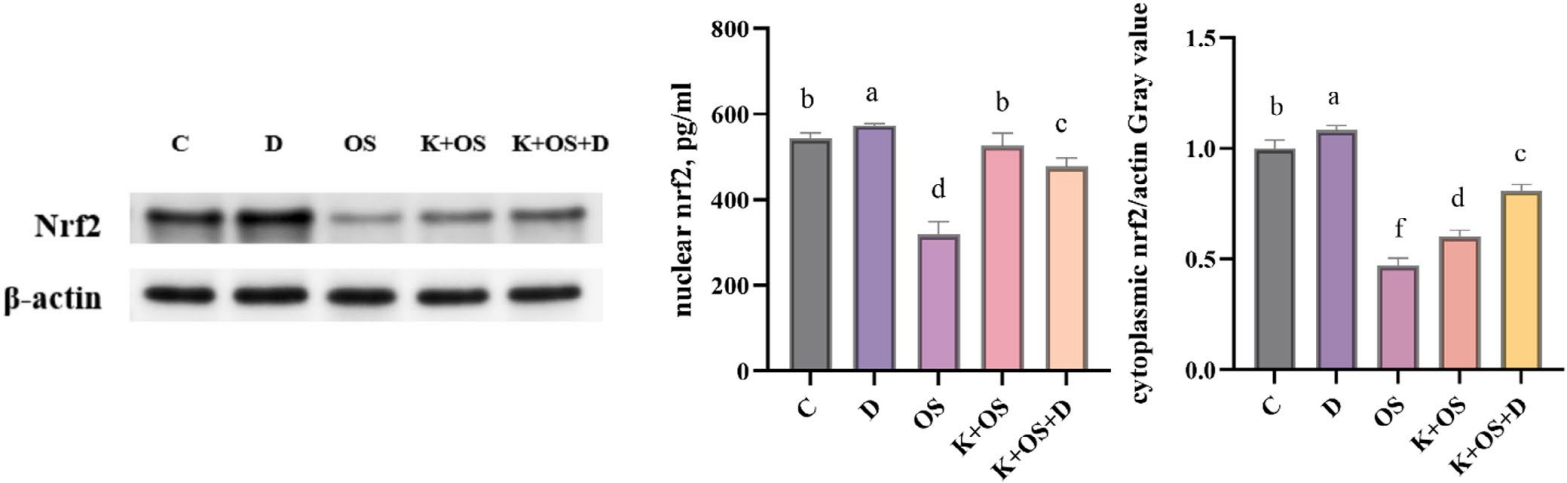
The effect of kaempferol on Nrf2 protein in the nucleus and cytoplasm of PK‐15 cells after Nrf2 activator treatment. After seeding in 6‐well plates, PK‐15 cells were pretreated with 50 μM DMF (or vehicle) for 24 h, incubated with 50 μM kaempferol (or vehicle) for a further 24 h, and finally exposed to 1 mM H_2_O_2_ for 3 h. The results are presented as the mean ± SE, *n* = 6. Values without common letters (a–c) differ significantly (*p* < 0.05). D, DMF; K, kaempferol; OS, hydrogen peroxide.

## 4 Discussion

In animal production, flavonoid compounds are recognized for their significant roles in reducing the risk of infectious diseases, enhancing immune function, and improving growth performance (Chen, He, et al. 2025; Tan et al. 2022). In the present study, dietary supplementation with 400 mg/kg kaempferol‐enriched *Sophora Fructus* extract significantly reduced the feed‐to‐gain ratio and increased body weight in piglets. These results indicate that kaempferol‐enriched *Sophora Fructus* extract exhibits a beneficial promotive effect on improving growth performance in piglets. Our findings align with previous research demonstrating the growth‐promoting effects of plant flavonoids. One study indicates that including high‐active isoflavone fermented soybean meal at an 8% inclusion rate in the diet significantly improved the growth performance of weaned piglets (Lingling and Jianxiong 2017). While some studies have indicated that plant flavonoids do not significantly enhance growth performance, other research has shown that they can increase the feed conversion ratio (Kamil et al. 2023). These discrepancies in our results may stem from factors such as feed composition, additive types, and rearing environments.

Our study found that dietary supplementation with 400 mg/kg kaempferol‐enriched *Sophora Fructus* extract significantly increased serum T‐AOC and GSH‐Px levels in piglets. Additionally, it showed an increasing trend in SOD and POD activities. These results suggest that kaempferol‐enriched *Sophora Fructus* extract enhances growth performance in piglets, likely by boosting antioxidant capacity and reducing oxidative stress damage. Existing studies confirm that plant‐derived bioactive components significantly elevate serum antioxidant enzyme activities and enhance overall antioxidant status (Wang et al. 2017). Previous research has shown that *Forsythia suspensa* extract significantly decreased serum MDA levels in piglets 14 days post‐weaning (Panfeng et al. 2017). Similarly, dietary supplementation with 6000 mg/kg hesperidin or naringin for 14 days significantly decreased the MDA content in ewe milk and improved its oxidative stability (Panagiotis et al. 2019). In 35‐day‐old meat rabbits, dietary addition of 0.02% seabuckthorn flavonoids significantly increased serum SOD activity and T‐AOC levels, while doses of 0.02%, 0.03%, and 0.04% all significantly decreased serum MDA content (Xinyu et al. 2023). Consistent with this, one study reported that *Eucommia ulmoides* flavonoids significantly increased serum SOD and GSH‐Px activities in weaned piglets induced by deoxynivalenol, decreased MDA and ROS levels, and improved growth performance (Yuan et al. 2022). These findings are consistent with those of the present study, which demonstrated that dietary supplementation with kaempferol‐enriched *Sophora Fructus* extract enhances antioxidant enzyme activities and thereby increases the antioxidant capacity of weaned piglets. The underlying mechanism likely involves the activation of the Nrf2 signaling pathway, leading to the suppression of ROS levels and a reduction in peroxidation products.

The Nrf2 signaling pathway is recognized as one of the core regulatory mechanisms for cellular defense against oxidative stress (Lu et al. 2025). Existing studies demonstrate that natural plant flavonoids can activate this pathway, enhance the expression of antioxidant enzymes, and thereby elevate the overall antioxidant capacity of cells. Studies have reported that quercetin attenuates diquat‐induced cell injury by promoting protein abundance of Nrf2 and regulating GSH‐related redox homeostasis in enterocytes (Hai et al. 2021). Studies have also shown that dietary supplementation with 100 mg/kg *Eucommia ulmoides* flavonoids upregulates the protein expression of Nrf2 and Keap1 in the jejunal mucosa of weaned piglets exposed to diquat, while also increasing the mRNA levels of *HO‐1* and *NQO1* and reducing oxidized glutathione (GSSG) concentration and the ratio of GSSG to GSH. This indicates that *Eucommia ulmoides* flavonoids exert antioxidant effects by activating the Nrf2 pathway and regulating downstream antioxidant enzymes (Dingfu et al. 2019). Furthermore, one study demonstrated that baicalin mitigates H_2_O_2_‐induced oxidative stress in IPEC‐J2 cells by activating the Nrf2/AMPK pathway (Jiahua et al. 2023). Another study using an LPS‐induced oxidative damage model showed that treatment with 16 μg/mL baicalin similarly attenuates oxidative damage in intestinal cells via the Nrf2/HO‐1 signaling axis (Yuefeng et al. 2021). Consistent with these findings, the present study revealed that supplementation with 400 mg/kg kaempferol‐enriched *Sophora Fructus* extract significantly increased the expression of Nrf2 and HO‐1 proteins while suppressing Keap1 protein expression in piglet serum. The underlying mechanism likely involves modulating the Nrf2 signaling pathway to enhance antioxidant capacity and reduce oxidative stress‐induced damage.

Given the critical role of oxidative stress in the porcine dermatitis and nephropathy syndrome (PDNS) in weaned piglets, we established an oxidative stress model in PK‐15 cells to explore the potential antioxidant effects of kaempferol.

Currently, ML385 is primarily utilized in research as an Nrf2 inhibitor to validate whether candidate drugs act as Nrf2 pathway agonists or to investigate their biological functions through the regulation of Nrf2 signaling (Hee et al. 2018; Wang et al. 2018; Xinnong et al. 2018). In this study, our findings revealed that in the presence of ML385, kaempferol failed to restore the expression of Nrf2 and its downstream genes, indicating that its effects depend on the Nrf2 signaling pathway. Conversely, the combination of DMF and kaempferol significantly upregulated the expression of Nrf2, HO‐1, and NQO1 while suppressing Keap1, demonstrating a synergistic effect between them. These results are consistent with previous studies by Lamberti and Kim (Lamberti et al. 2018; Oh et al. 2016), which reported that kaempferol exerts its antioxidant effects through the Nrf2/ARE pathway. Additionally, the combination of DMF and kaempferol significantly reduced cytoplasmic Nrf2 protein expression but significantly increased nuclear Nrf2 expression. Collectively, these results suggest that kaempferol may enhance cellular antioxidant capacity and exert defensive effects against oxidative stress by activating the Nrf2 signaling pathway and regulating the expression of related upstream and downstream genes and proteins.

Several limitations of the present study should be acknowledged. The animal experiment included only 6 piglets per treatment, with each piglet serving as one experimental unit. The small sample size limits the statistical power and precision of the estimated treatment effects and may restrict the generalizability of the findings. Therefore, the observed effects on growth‐related outcomes should be considered preliminary and require confirmation in larger, adequately powered feeding trials.

## 5 Conclusion

In conclusion, dietary supplementation with 400 mg/kg kaempferol‐enriched *Sophora Fructus* extract was associated with improved growth performance and enhanced antioxidant capacity in weaned piglets. Kaempferol also exerted protective effects against H_2_O_2_‐induced oxidative stress in vitro. The mechanism by which kaempferol enhanced antioxidant capacity may be related to the activation of the Nrf2 signaling pathway.

## Author Contributions

**Yanhui Liang:** conceptualization, software, formal analysis, validation, writing – original draft. **Yan Liu:** methodology. **Leqi Tong:** methodology. **Xinyue Fu:** methodology, software, formal analysis. **Zhijian Du:** conceptualization, data curation, formal analysis, validation, writing – original draft, writing – review and editing. **Jing Zhang:** conceptualization, data curation, writing – original draft, writing – review and editing. **Qinghua Liu:** conceptualization, data curation, writing – review and editing.

## Funding

This research was funded by the National Key Research and Development Program of China, grant number 2023YFD1600502 and Horizontal Funding Project of Fujian Agriculture and Forestry University (FAFU) (Grant No. KH260091A).

## Ethics Statement

The animal study was reviewed and approved by the Animal Care and Use Committee of Fujian Agriculture and Forestry University (Approval ID: PZCASFAFU24046).

## Consent

The authors have nothing to report.

## Conflicts of Interest

The authors declare no conflicts of interest.

## Data Availability

The data that support the findings of this study are available from the corresponding author upon reasonable request.
